# Supplementary material for: INO80 Is Required for the Cell Cycle Control, Survival, and Differentiation of Mouse ESCs by Transcriptional Regulation
Source: Int J Mol Sci. 2022 Dec 6;23(23):15402. doi: 10.3390/ijms232315402 (PMC9740483; doi:10.3390/ijms232315402)
Supplement: Supplementary file 1 [file ijms-23-15402-s001.zip › supplemetary_figure.pdf]

Cell cycle analysis in ESC (related to Figure 1G)

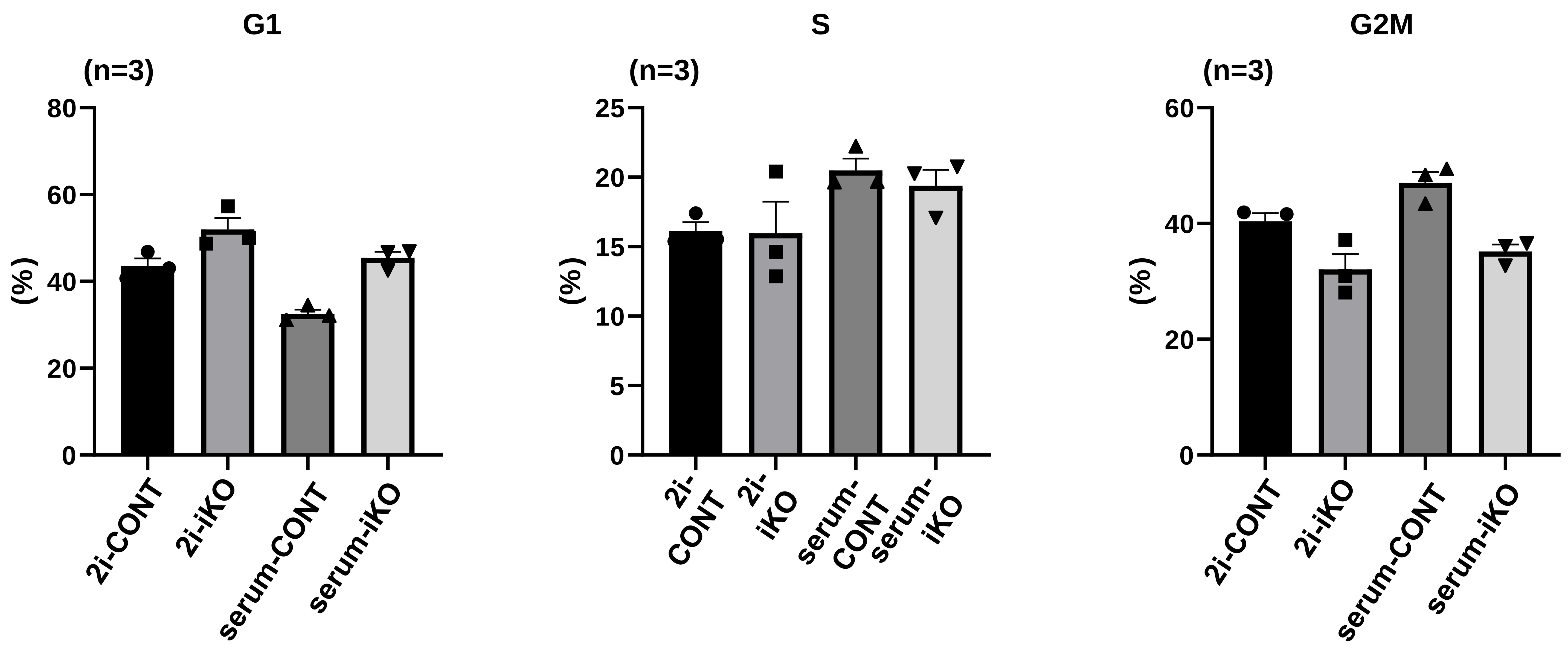

Figure S1. Cell cycle analysis in ESC.

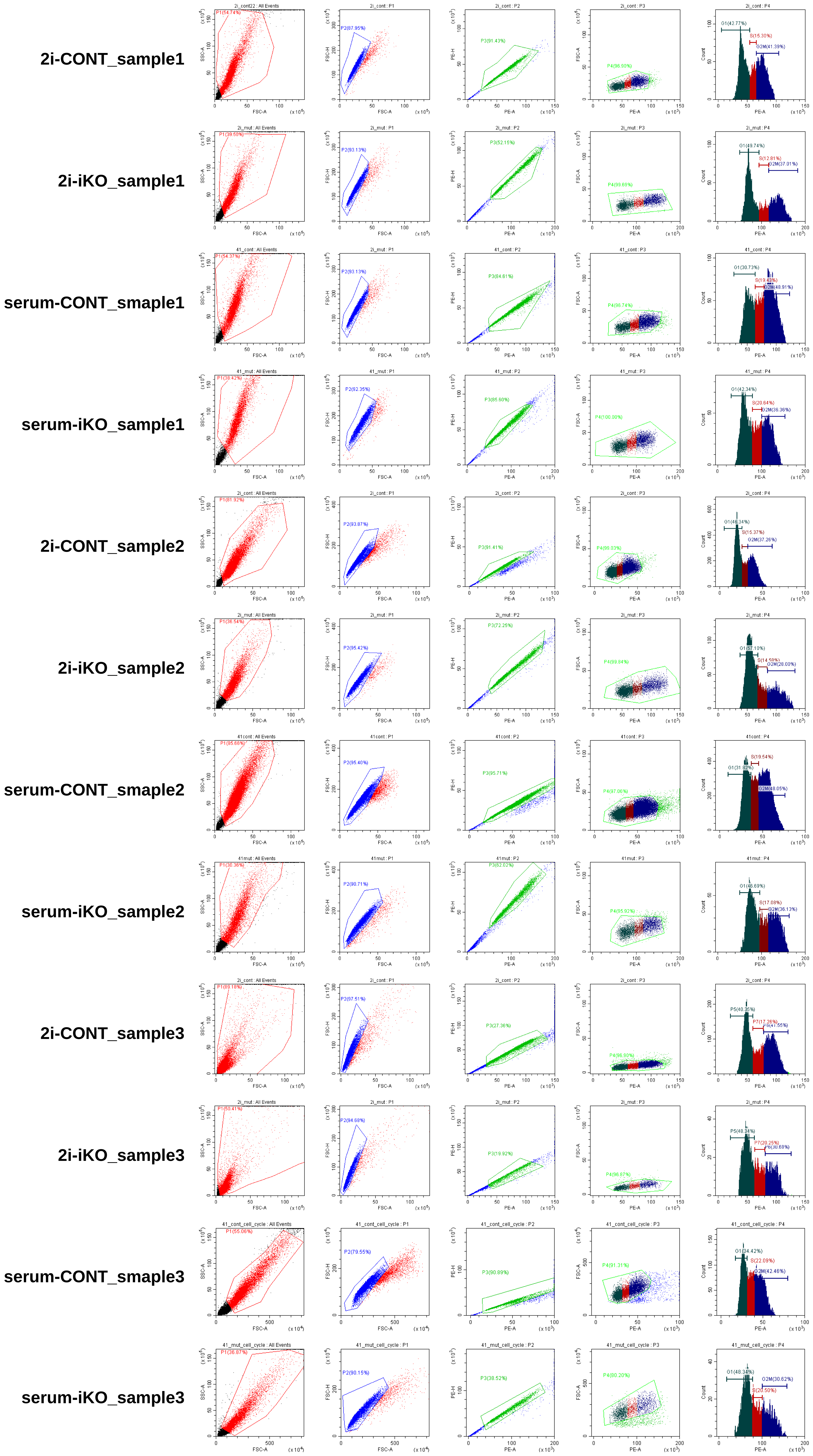

Figure S2. Panels of flow cytometric analysis of ESC.

Cell cycle analysis in differentiating ESC (related to Figure 3C)

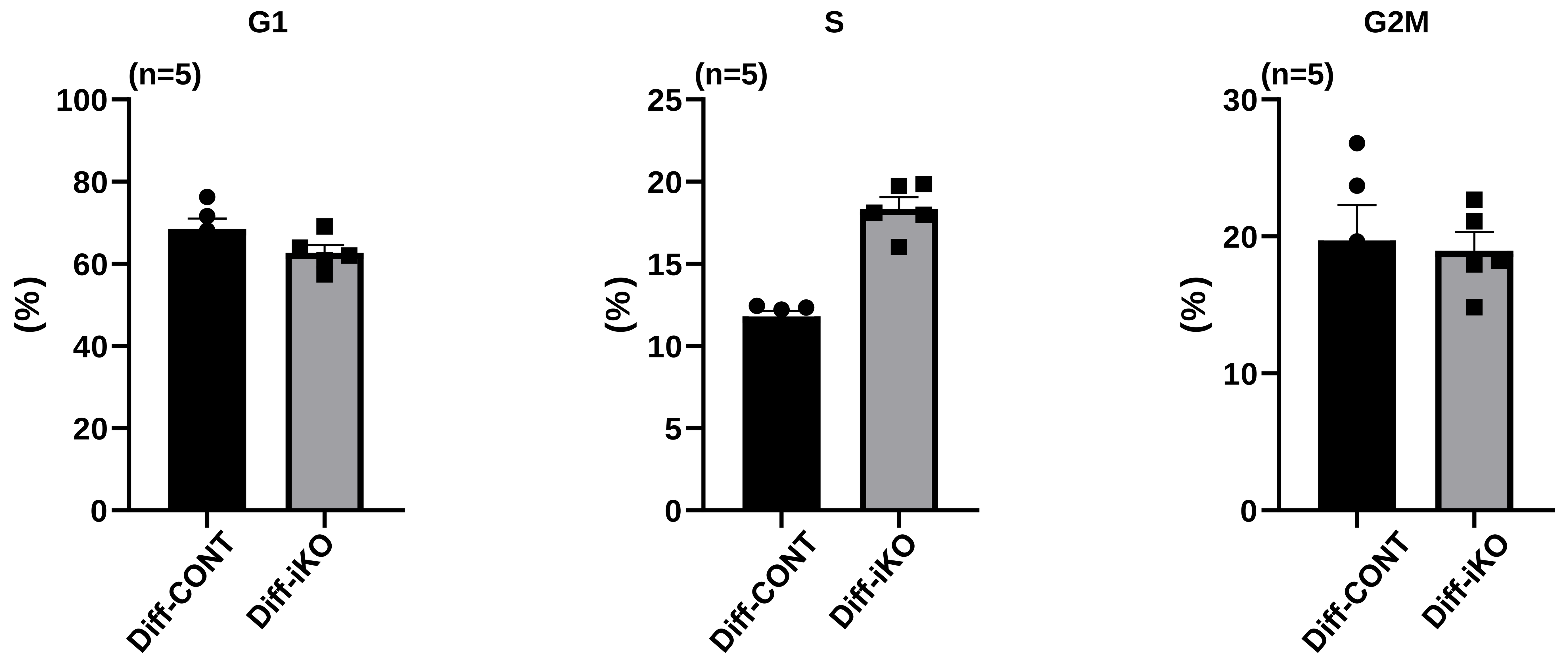

Figure S3. Cell cycle analysis in differentiating ESC.

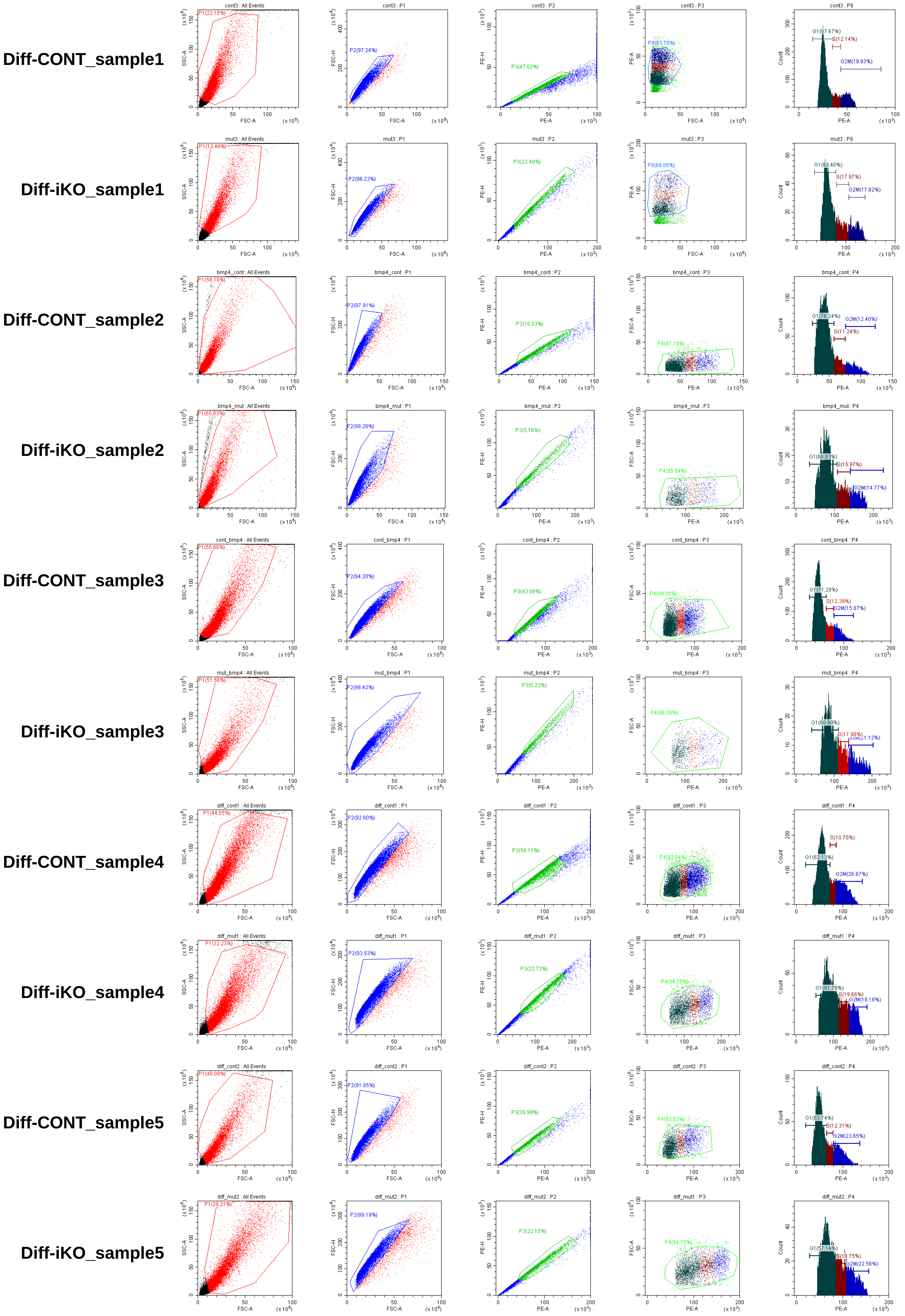

Figure S4. Panels of flow cytometric analysis of differentiating ESC.
